# Nuclear-cytoplasmic asynchrony in oocyte maturation caused by TUBB8 variants via impairing microtubule function: a novel pathogenic mechanism

**DOI:** 10.1186/s12958-023-01161-y

**Published:** 2023-11-22

**Authors:** Tianli Chang, Jing Zhao, Qi Li, Anning Meng, Qiuping Xia, Yanping Li, Wenpei Xiang, Zhongyuan Yao

**Affiliations:** 1 Reproductive Medicine Center, Xiangya Hospital, Central South University, Changsha, 410078 Hunan China; 2 Institute of Reproductive Health, Center of Reproductive Medicine, Tongji Medical College, Huazhong University of Science and Technology, Hongshan, China

**Keywords:** TUBB8, Oocyte, Spindle, Mitochondria, Endoplasmic reticulum, Microtubules, Meiosis

## Abstract

**Background:**

TUBB8, a crucial gene encoding microtubule protein, plays a pivotal role in cellular processes. Deleterious TUBB8 variants have been shown to significantly hinder oocyte maturation. In this study, we conducted an in vitro investigation using TUBB8 mutant mouse oocytes to elucidate the pathogenic mechanisms of TUBB8 variants in oocyte nuclear and cytoplasmic maturation.

**Methods:**

A mutant model was successfully established in mouse oocytes via microinjection to further investigate the effects of four novel discovered TUBB8 mutations on the nuclear and cytoplasmic maturation of mouse oocytes. Immunofluorescence and confocal microscopy were performed to observe the cortical polarity and spindle and of mutant oocytes. Active mitochondrial staining was performed to analyze mitochondrial distribution patterns. Endoplasmic reticulum and Ca^2+^ staining were conducted to assess ER distribution and cytoplasmic calcium ion concentration in oocytes.

**Results:**

In mouse oocytes, TUBB8 variants (p.A313V, p.C239W, p.R251Q, and p.G96R) resulted in a reduction of the first polar body extrusion rate, disruption of spindle assembly, and abnormal chromosome distribution. Additionally, these variants induced oocyte organelle abnormalities, including anomalies in mitochondrial redistribution and endoplasmic reticulum stress compared to the wild-type.

**Conclusion:**

Deleterious TUBB8 variants could disrupt microtubule function, affecting critical processes such as spindle assembly, chromosome distribution, and organelle rearrangement during oocyte meiosis. These disruptions culminate in compromised nuclear-cytoplasmic maturation, consequently giving rise to oocyte maturation defects.

## Background

The maturation of oocytes is an important prerequisite for fertilization and embryo development. Oocyte maturation defects are the pathological phenotypes of primary infertility, characterized by the inability to obtain mature oocytes through either ovulation induction treatment or in vitro oocyte maturation [1]. Oocyte maturation involves a series of morphological and molecular changes, which are tightly regulated by genes [2]. With the development and widespread application of genetic sequencing technologies, elucidating the genetic and molecular regulatory mechanisms underlying oocyte maturation disorders at the microscopic gene level holds significant practical significance for exploring rational intervention strategies.

TUBB8 is a gene that encodes a subunit of microtubule protein and is the first gene discovered to be associated with oocyte maturation arrest. It is involved in encoding an important subunit of microtubule protein. Microfilaments and microtubules are the main components of the cellular cytoskeleton [3]. The actin filament network in the cell cortex, which is composed of microfilaments, determines cell shape and polarity. Microtubules, the major components of the cell skeleton, are assembled from dimers of α and β tubulin proteins [4]. Proper distribution of meiotic genetic material, migration and arrangement of cytoplasmic organelles, and accumulation of maternal substances during oocyte maturation highly rely on the regulation of the microtubule system.

Oocyte maturation involves nuclear maturation and cytoplasmic maturation. Nuclear maturation of the oocyte refers to the completion of correct meiotic division and the extrusion of the first polar body. Proper assembly of the spindle and chromosome alignment are crucial for nuclear maturation. During meiotic division, processes such as chromosome movement and distribution, spindle formation and rotation, and kinetochore microtubule attachment rely heavily on microtubule protein dynamics [5]. Thus, microtubule proteins play a vital role in ensuring orderly meiotic division. However, the extrusion of the first polar body by the oocyte does not guarantee its developmental competence. Synchronous nuclear and cytoplasmic maturation is the true definition of oocyte maturation, which ensures the oocyte’s fertilization and developmental potential [6–8]. Cytoplasmic maturation of the oocyte refers to the coordinated spatial distribution of organelles and the accumulation of substances within the organelles, including energy reserves, enzyme reserves, and protein synthesis reserves. Cytoplasmic maturation provides the material foundation for fertilization and early embryonic development after ovulation and plays a crucial role in oocyte function and embryo development, which is also a significant reason for the large size of oocytes [9]. Importantly, microtubule proteins play an indispensable role in the rearrangement of mitochondria and endoplasmic reticulum distribution in the oocyte during this process [10–12]. Mitochondria are the most abundant organelles in the cytoplasm of the oocyte, and the ATP they produce serves as the direct energy source for oocyte meiotic division and early embryonic development [13]. The endoplasmic reticulum serves as a site for the storage and release of calcium ions in the oocyte and plays a crucial role in regulating calcium ion signaling within the cell [14].

Previous studies have identified the pathogenicity of multiple TUBB8 variants in the blockage of oocyte maturation. In 2016, Wang et al. disclosed that TUBB8 variants caused microtubule disruption on expression in cultured cells and in oocytes. This made a dominant and cumulative effect when a critical proportion of mutant heterodimers are incorporated into polymers, leading to greater instability, as compared with microtubules in wild-type cells, and frequently to complete annihilation of microtubules [15, 16]. More TUBB8 variants were reported from patients with oocyte maturation arrest that these deleterious variants affected the formation and stability of the β-tubulin isotype, disrupted spindle morphology, and subsequently hinder the process of oocyte meiotic division, ultimately leading to oocyte maturation defects [16–18]. Some variants are associated with new phenotypes, such as large polar body oocyte, fertilization failure and Multiple pronuclei (MPN) formation [19, 20]. However, TUBB8 as a gene which encodes a β-tubulin isotype, the impact of TUBB8 variants causing microtubule system functional deficiencies on the cytoplasmic organelle maturation of oocytes has not been reported.

In this study, we focused on the effect of TUBB8 variants on microtubule function during the nuclear and cytoplasmic maturation of mouse oocytes. We discovered a novel pathogenic effect of TUBB8 variants in oocyte maturation in vitro and our findings further expand the spectrum of TUBB8 variations associated with oocyte maturation defects, potentially providing targets for future therapeutic strategies.

## Results

### TUBB8 variants hampered the extrusion of first polar body in mouse oocytes

**Fig. 1:**
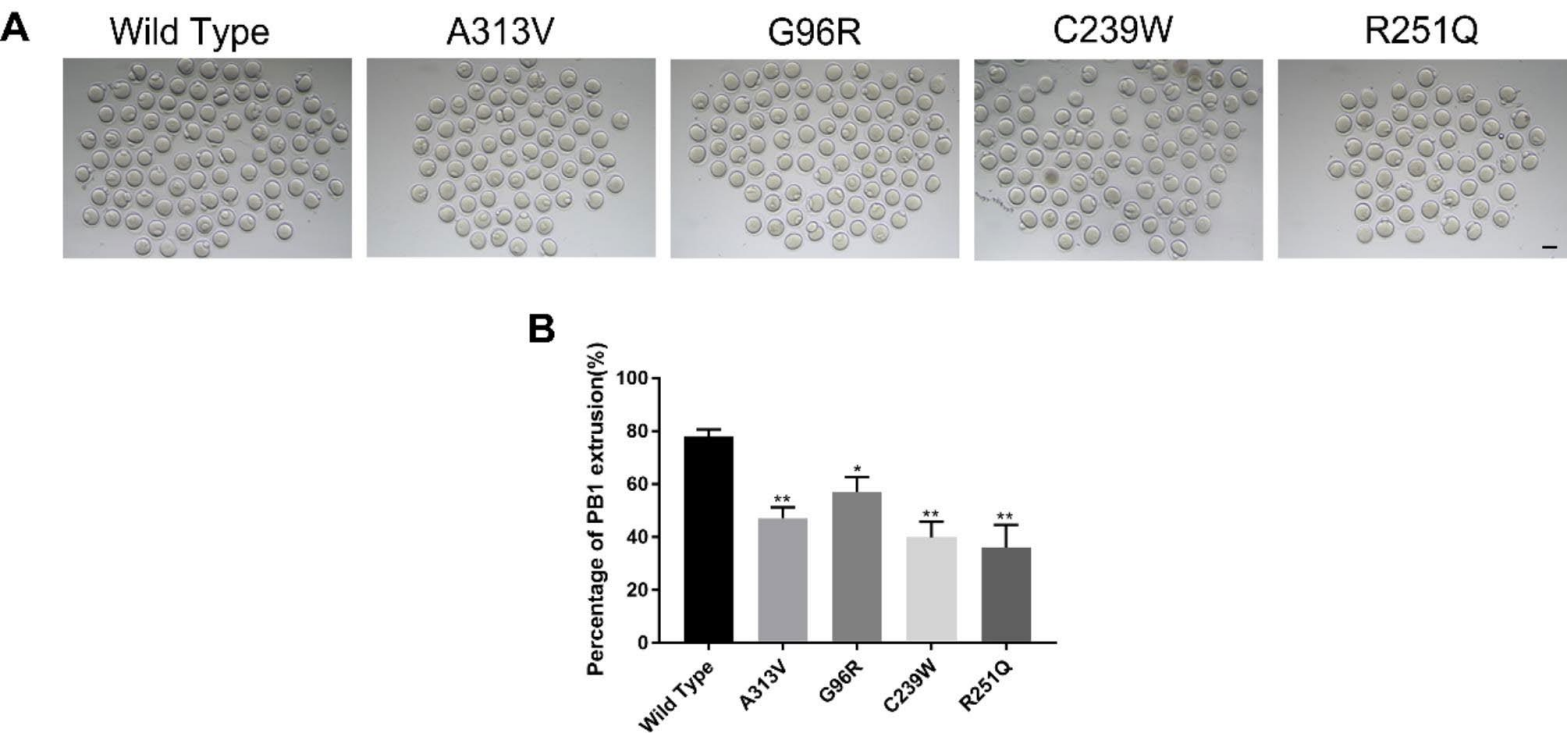
The rate of extrusion of the first polar body in mouse oocytes with TUBB8 variants. (**A**) Representative images of in vitro matured oocytes. (**B**) Statistics of the rate of first polar body extrusion in mouse oocytes in vitro. Data are presented as mean ± standard deviation (mean ± SD), *P < 0.05; **P < 0.01

In order to investigate the potential impact of various TUBB8 variants on the in vitro maturation process of oocytes, we performed microinjections of WT and four mutant TUBB8 mRNA into mouse oocytes in vitro. The results revealed a significant reduction in the rate of first polar body extrusion in the p.A313V, p.G96R, p.C239W, and p.R251Q mutant TUBB8 groups compared to the WT group (Fig. 1A/B, 77.86% ± 2.80%, n = 152, vs. 47.08% ± 4.17%, n = 138, 56.93% ± 5.80%, n = 144, 39.97% ± 5.71%, n = 147, 36.14% ± 8.38%, n = 167, *P < 0.05; **P < 0.01). These findings suggested that TUBB8 variants led to a decrease in the extrusion rate of the polar body during the in vitro oocyte maturation process.

### TUBB8 variants led to abnormal spindle morphology in mouse oocytes

**Fig. 2:**
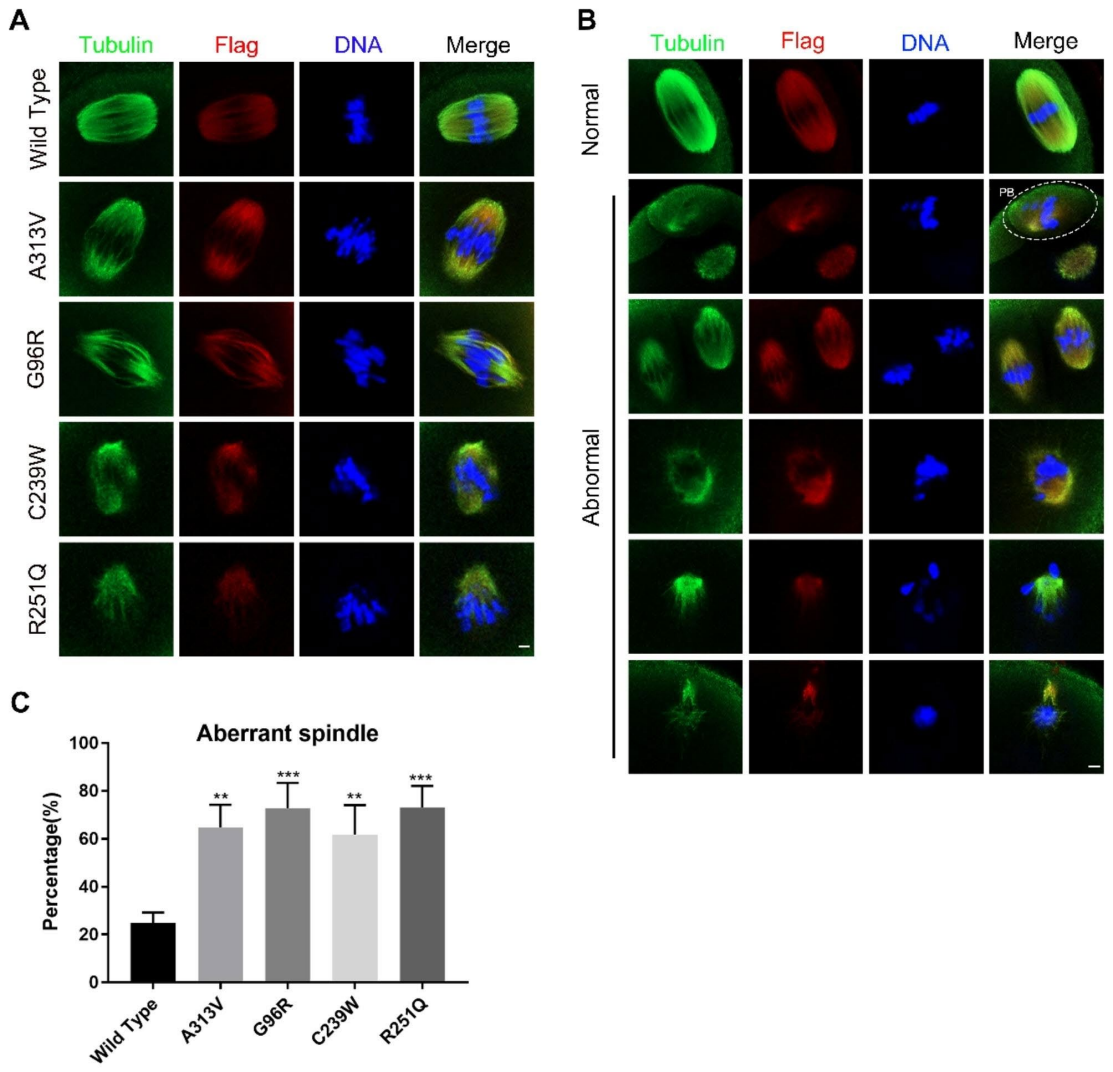
TUBB8 variants lead to abnormal spindle morphology in mouse oocytes. (**A**) Representative images of spindle morphology in mouse oocytes at the metaphase II (MII) stage, with Flag indicating the signal of exogenous protein transduction, scale bar = 5 μm. (**B**) Abnormal spindle types in mouse oocytes at the MII stage, with Flag indicating the signal of exogenous protein transduction, and the white dashed box indicating the first polar body, scale bar = 5 μm. (**C**) Statistical analysis of the percentage of abnormal spindles in mouse oocytes. Data are presented as mean ± standard deviation (mean ± SD), **P < 0.01; ***P < 0.001

Microtubule proteins are the main constituents of oocyte spindles, and TUBB8 is an important gene encoding microtubule protein. After microinjection of WT and four mutant TUBB8 mRNA into mouse oocytes in vitro, we found that oocytes in the p.A313V, p.G96R, p.C239W, and p.R251Q variant groups exhibited varying degrees of spindle abnormalities. In the A313V group, the spindle fibers showed no obvious abnormalities, while in the G96R group, the spindle fibers were disorganized, with uneven distribution and asymmetrical polarity. The C239W group showed dispersed spindle fibers, losing polarity, and the R251Q group exhibited a loss of normal spindle structure (Fig. 2A). Furthermore, we observed the presence of abnormal spindle types in all groups, such as spindles lacking proper structure, multiple spindle structures, multipolar spindles, short spindles, and asymmetric spindles (Fig. 2B). Statistical analysis revealed a significant increase in the percentage of abnormal spindles in the p.A313V, p.G96R, p.C239W, and p.R251Q mutant TUBB8 groups compared to the WT group (Figs. 2C and 24.67% ± 4.51%, n = 39, vs. 64.67% ± 9.50%, n = 51, 72.67% ± 10.69%, n = 45,61.67% ± 12.34%, n = 47, 73.00% ± 9.165, n = 49, **P < 0.01; ***P < 0.001.).

### TUBB8 variants induced aberrant chromosome distribution in mouse oocytes

**Fig. 3:**
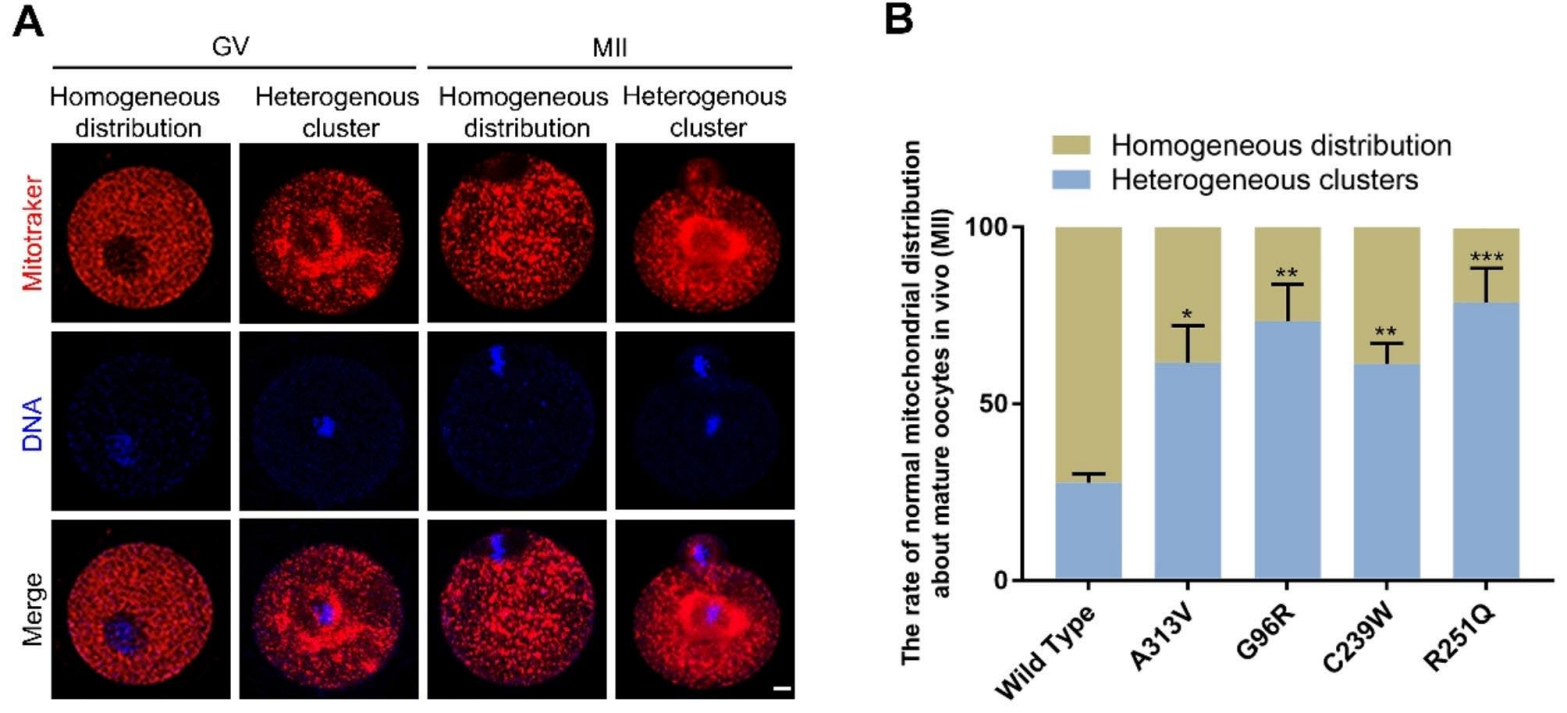
Changes in mitochondrial distribution in mouse oocytes caused by TUBB8 variants (**A**) Mitochondrial distribution patterns in mouse oocytes during GV and MII stages, scale bar = 10 μm. (**B**) Statistical analysis of mitochondrial distribution proportions in mouse oocytes at the MII stage; data are presented as mean ± standard deviation (mean ± SD), *P<0.05; **P<0.01; ***P<0.001

**Fig. 4:**
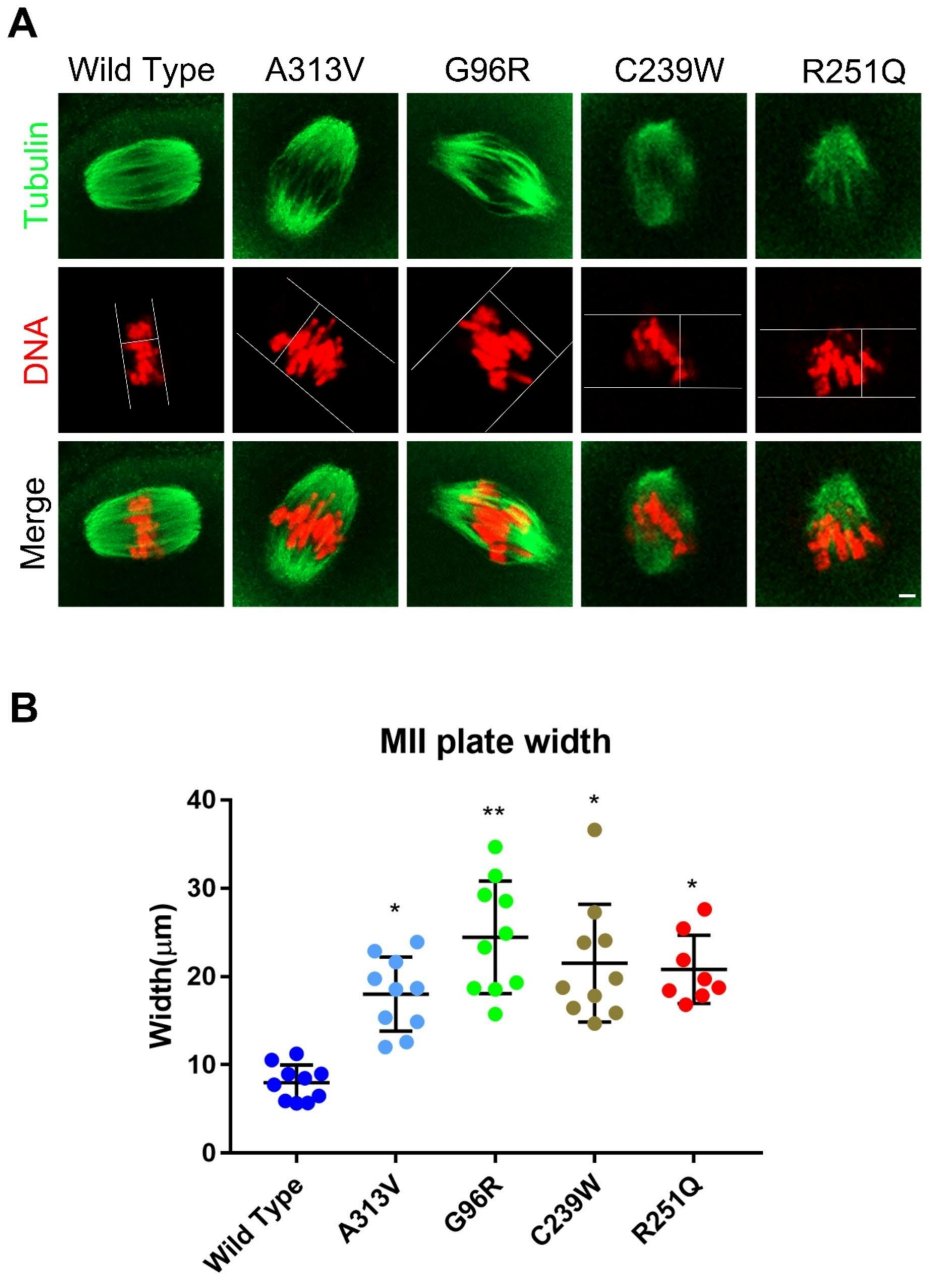
Abnormal chromosome distribution in mouse oocytes caused by TUBB8 variants. (**A**) Chromosome plate structure in the spindles of mouse oocytes at the metaphase II (MII) stage, scale bar = 5 μm. (**B**) Statistical analysis of chromosome plate width in the spindles of mouse oocytes at the MII stage. Data are presented as mean ± standard deviation (mean ± SD), *P < 0.05; **P < 0.01

To further explore the impact of TUBB8 variants on chromosome distribution in oocyte spindles, we analyzed the chromosome plate structure in the spindles of each group of oocytes (Fig. 3A). The results showed that compared to the WT group, the p.A313V, p.G96R, p.C239W, and p.R251Q mutant TUBB8 groups exhibited a significant increase in the longitudinal axis width of the chromosome plate in the metaphase II (MII) oocyte spindles (Fig. 4A/B, 7.96 ± 2.02, n = 42, vs18.02 ± 4.19, n = 50, 24.44 ± 6.365, n = 47, 21.52 ± 6.68, n = 49, 20.81 ± 3.87, n = 50, *P < 0.05; **P < 0.01.). This suggested that the various TUBB8 variants affected the arrangement, distribution density, and dynamics of microtubule proteins in the spindle structure, leading to abnormal chromosome plate distances. This uneven distribution of chromosomes can further result in the occurrence of oocyte aneuploidy.

### TUBB8 variants did not affect the cortical polarity of mouse oocytes

**Fig. 5:**
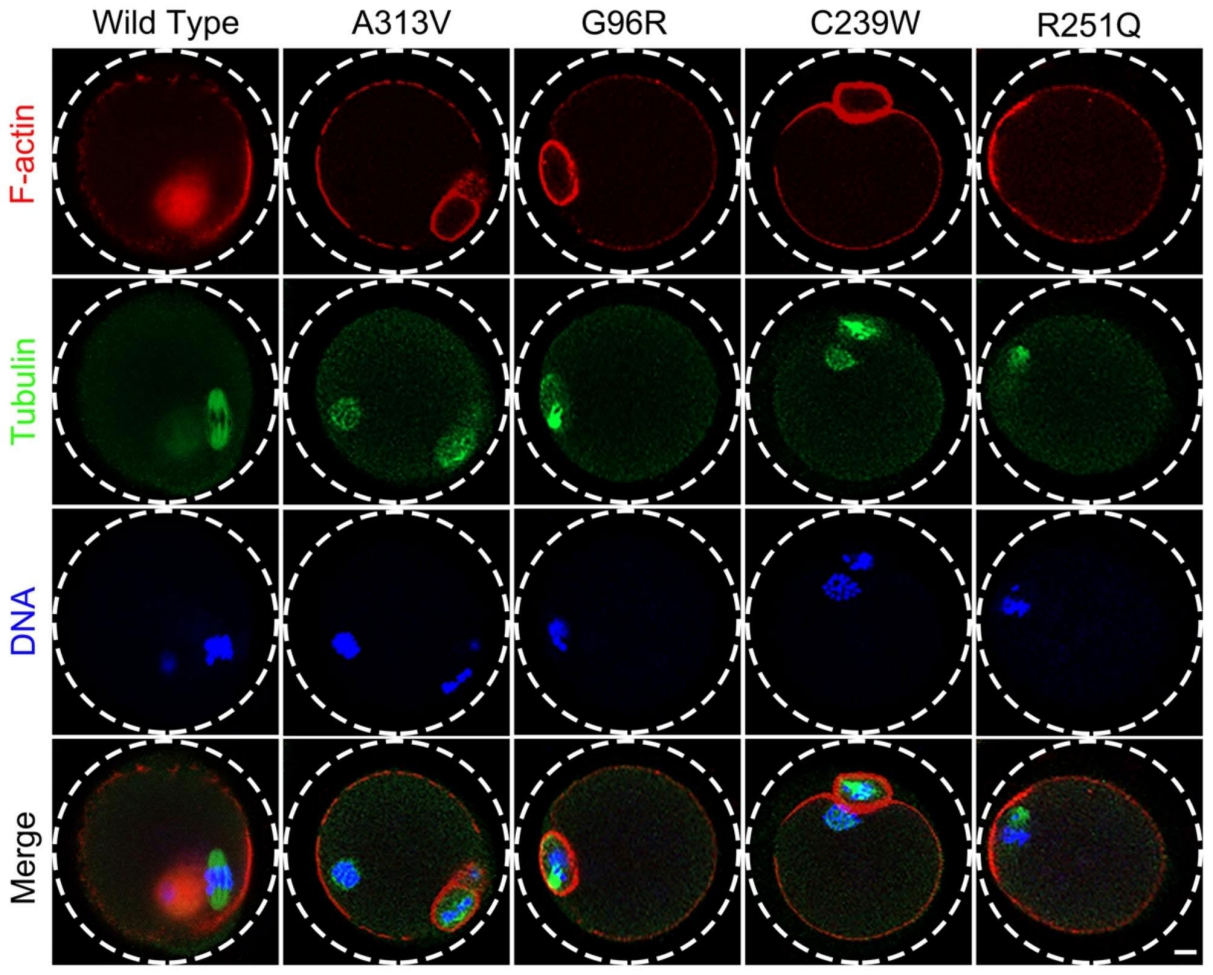
Formation of the cortical actin cap in mouse oocytes. F-actin represents the cortical actin cap structure in oocytes, scale bar = 10 μm

The haploid oocyte generation process involves meiotic and asymmetric cell division. The polarity of oocytes determines the formation position and size of the two polar bodies, ensuring the production of haploid oocytes while retaining almost all cytoplasm within the oocyte for early embryonic development [21–23].The polarity of oocytes is crucial for proper meiotic division and spindle movement, and it is an important manifestation of oocyte maturation. Immunofluorescence staining of oocytes in different groups revealed that both wild-type (WT) and TUBB8 mutant oocytes at the metaphase II (MII) stage can form normal F-actin caps (Fig. 5), indicating that the TUBB8 variants do not affect the polarity of mature oocytes.

### TUBB8 variants disrupted mitochondrial distribution in mouse oocytes

Two distribution patterns of mitochondria are observed during oocyte maturation: clustered and uneven distribution, and uniform distribution (Fig. 3A). The overall movement and distribution pattern of mitochondria in oocytes is from the vicinity of the nucleus toward the cortex, and the uniform distribution of mitochondria represents a higher level of cytoplasmic maturation in oocytes. The distribution and rearrangement of mitochondria are mainly achieved through the pulling action of microtubule proteins. To investigate whether TUBB8 variants affect the rearrangement of organelles during oocyte maturation, we performed live staining of mitochondria in WT and TUBB8 variant oocytes. The results showed that the proportion of oocytes with uniformly distributed mitochondria at the metaphase II (MII) stage was significantly decreased in the p.A313V, p.G96R, p.C239W, and p.R251Q TUBB8 mutant groups compared to the WT group (Figs. 3B and B and 72.33 ± 2.51, n = 58, vs. 38.33 ± 10.40, n = 49, 26.67 ± 10.41, n = 50, 38.67 ± 5.69, n = 53, 21.00 ± 9.54, n = 55, *P<0.05; ** *P*<0.01;*** *P*<0.001). This indicates that the TUBB8 variants cause rearrangement and redistribution of mitochondria during oocyte maturation, leading to a decrease in cytoplasmic maturation of oocytes.

### TUBB8 variants triggered an endoplasmic reticulum (ER) stress response

**Fig. 6:**
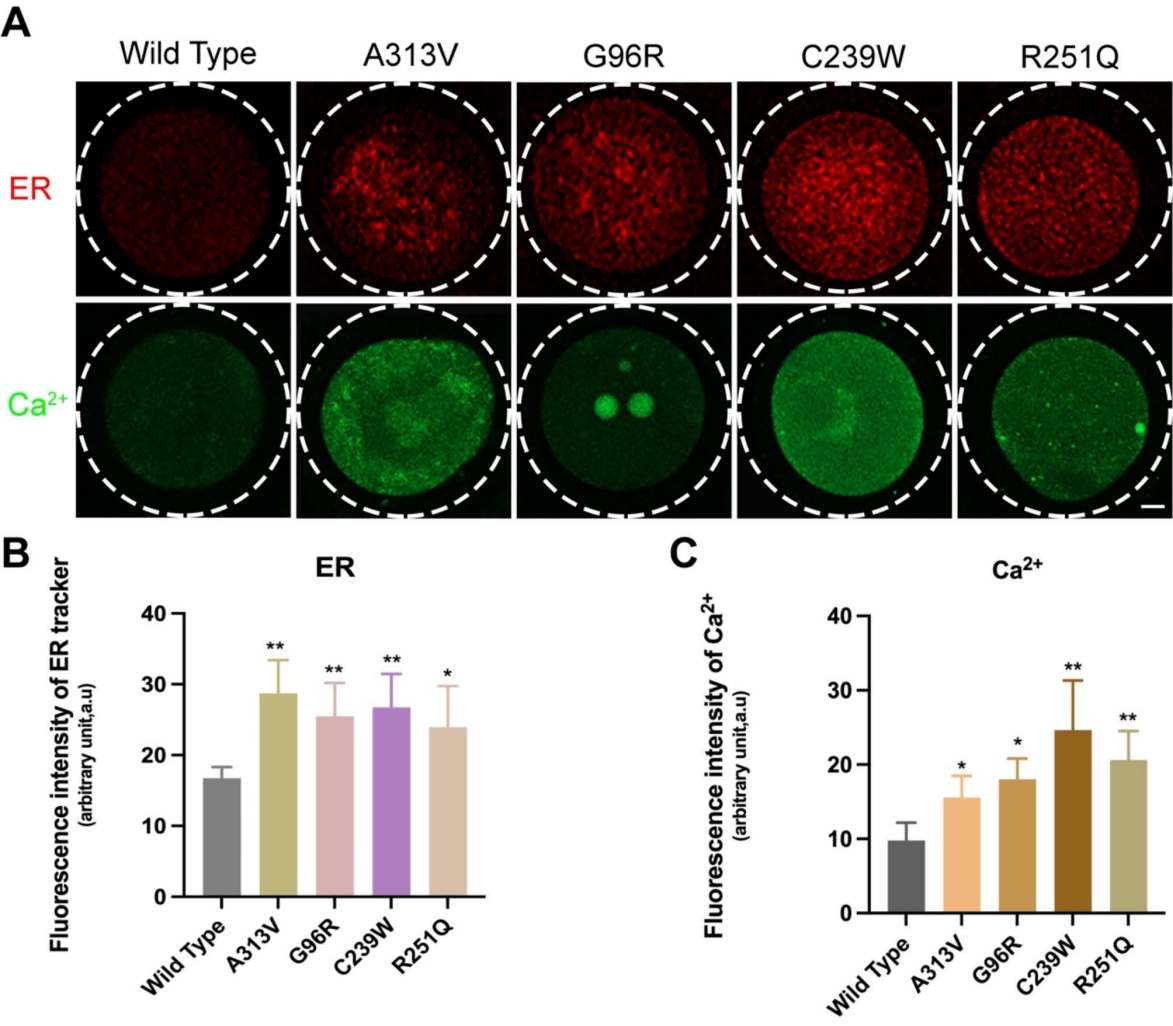
TUBB8 variants induces endoplasmic reticulum (ER) stress response in mouse oocytes. (**A**) Fluorescence images showing the distribution of ER in oocyte cytoplasm; fluorescence images depicting the distribution of calcium ions in oocytes. Scale bar = 10 μm. (**B**) Analysis of ER fluorescence intensity in oocytes. (**C**) Analysis of calcium ion fluorescence intensity in oocytes. Data are presented as mean ± standard deviation (mean ± SD), *P<0.05; ** *P*<0.01

We further examined the distribution of the endoplasmic reticulum (ER) and the calcium ion concentration in the cytoplasm of oocytes. The results revealed an increased content of ER (Fig. 6A/B, 16.75 ± 1.56, n = 45, vs. 28.71 ± 4.69, n = 53, 25.48 ± 4.67, n = 50, 26.73 ± 4.73, n = 52, 23.94 ± 5.80, n = 47, *P<0.05; ** *P*<0.01) and an elevated calcium ion concentration (Fig. 6A/C, 9.77 ± 2.43, n = 54, vs. 15.59 ± 2.90, n = 59, 18.02 ± 2.80, n = 52, 24.65 ± 6.68, n = 48, 20.61 ± 3.92, n = 51, *P<0.05; ** *P*<0.01), indicating impaired ER calcium storage and release capacity, leading to ER stress. These findings suggest an endoplasmic reticulum (ER) stress response, a reduced cytoplasmic maturation and compromised developmental potential of oocytes.

## Discussion

Our team has identified four novel TUBB8 variants from clinical cases of oocyte maturation arrest. Among them, p.C239W, p.R251Q, and p.G96R variants were predicted to be deleterious, while the p.A313V1 mutation was predicted to be benign. We validated their pathogenicity in Hela cells through in vitro transfection, and the results were consistent with the predictions. Our previous study demonstrated that Hela cells transfected with p.C239W, p.R251Q, or p.G96R variants exhibited disrupted microtubule structures, while the p.A313V variant showed a similar abnormality rate of microtubule structure to the wild type (WT) [17]. The expression pattern was consistent with the predicted outcomes. In this study, a mutant model was successfully established in mouse oocytes through microinjection, which outperformed somatic cell models by better reflecting the occurrence of oocyte maturation arrest. This model enabled us to further investigate the effects of four novel discovered TUBB8 variants on the nuclear and cytoplasmic maturation of mouse oocytes.

Intriguingly, this study identified four novel deleterious TUBB8 variants during oocyte maturation in vitro (p.A313V, p.C239W, p.R251Q, and p.G96R). The exclusion rates of the first polar bodies in oocytes carrying these variants were significantly reduced compared to the wild-type, with most of them arrested at the MI stage, indicating the detrimental effects of p.A313V, p.C239W, p.R251Q, and p.G96R mutant TUBB8 in oocytes. Furthermore, we investigated the pathological mechanisms of oocyte mutations caused by the TU888 mutation. TUBB8 variants led to a significant increase in the longitudinal axis width and asymmetric distribution of spindle chromosomes in oocytes, indicating altered spindle dynamics. The mutation may cause changes in microtubule protein dynamics and lead to abnormal spindle structures such as double spindles, missing spindles, and multipolar spindles, with a significantly increased occurrence rate. Furthermore, we examined spindle structures. The p.A313V and p.G96R variants resulted in scattered arrangement of spindle fibers, uneven distribution of microtubule proteins, and asymmetric polarity. The p.C239W variant caused spindle fiber dispersion and loss of polarity, while the p.R251Q variant led to the loss of normal spindle structures in oocytes.

The nuclear maturation of oocytes relies on the combined action of multiple biological processes, including the formation of the spindle apparatus, equal distribution of chromosomes, and extrusion of the first polar body. In the spindle apparatus of metaphase II (MII) oocytes, homologous chromosomes are symmetrically aligned at the equatorial plate under the traction of spindle fibers. Abnormal spindle assembly and dynamics can lead to an asymmetric distribution of chromosomes during the second meiotic division, resulting in uneven distribution and asynchronous movement of chromosomes. This greatly increases the probability of non-disjunction in oocytes and consequently leads to fertilization failure and an increased risk of producing embryos with abnormal chromosomes. Therefore, although the oocytes with the four variants show the extrusion of the first polar body morphologically, their nuclei remain in an immature state. Additionally, the polarity of oocytes is crucial for the proper spindle movement and asymmetric division during normal meiosis, representing an essential aspect of oocyte maturation. In the cellular cytoskeleton, microfilaments, microtubules, and myosin proteins cooperate and complement each other in their occurrence, assembly, and function. Through immunofluorescent staining of oocytes from different groups, we found that both wild-type (WT) and TUBB8 mutant oocytes could form a normal actin cap (F-actin) during the MII stage, indicating that TUBB8 mutation does not affect the functions of microfilaments and myosin proteins.

The nuclear maturation of oocytes is accompanied by a series of organelle movements and biochemical reactions in the cytoplasm to ensure synchronous maturation of the cytoplasm and nucleus, thereby providing sufficient material preparation for normal fertilization and embryo development [8, 24]. During oocyte maturation, the migration and distribution of mitochondria and endoplasmic reticulum depend on the dynamics of microtubule proteins. The activation and redistribution of mitochondria are important features of cytoplasmic maturation. Mitochondrial rearrangement is closely associated with oocyte maturation, and mitochondria adjust their quantity and distribution in different regions to meet the energy demands of different stages of meiosis [25]. Oocytes entering meiosis increase their mitochondrial numbers to meet the energy demands of fertilization and early embryonic development. The general pattern of mitochondrial distribution changes during oogenesis and maturation is that mitochondria initially migrate towards the cortex of the oocyte. This is because active substance exchange and secretion of the zona pellucida between oocytes and cumulus cells require a significant amount of energy. As the oocyte matures, mitochondria gradually migrate from the cortical region to the central cytoplasmic region. Mitochondria in the ooplasm exhibit clustered distribution (including peripheral and semi-peripheral distributions) as well as uniform distribution [26]. In germinal vesicle-stage oocytes, mitochondria remained in the cortex of the oocyte that leads to poor embryo development after IVF. However, mitochondria relocated during maturation, so that in mature (metaphase II) oocytes, two distinct patterns of mitochondrial distribution were observed. Notably, when oocytes were matured in a culture medium enriched with glucose and amino acids, mitochondria primarily relocated to the central region of the oocyte, eventually culminating in a uniform distribution in mature (metaphase II) oocytes, facilitated by microtubule-driven transport. This particular distribution pattern was associated with oocytes displaying competence in blastocyst formation following IVF [27]. The observed pattern and mitochondrial localization appear to be closely linked to developmental competence. Our experimental results showed a significant decrease in the proportion of MII oocytes with uniformly distributed mitochondria in TUBB8 mutant groups (p.A313V, p.G96R, p.C239W, and p.R251Q) compared to the wild-type (WT) group. This suggested that TUBB8 variants cause a rearrangement of mitochondria distribution during oocyte maturation, leading to a decrease in cytoplasmic maturation.

The endogenous reactions of the endoplasmic reticulum (ER) and calcium homeostasis in the cytoplasm are crucial for oocyte maturation. The endoplasmic reticulum (ER) undergoes redistribution and structural changes during oocyte maturation, facilitated by the action of microtubule proteins [28]. We examined the distribution of ER and Ca2 + in MII-stage oocytes and found that the mutant group exhibited smooth endoplasmic reticulum (ER) clustering, increased ER content with uneven distribution, and significantly elevated Ca2 + concentration (Fig. 6A/B). These observations suggest impaired storage and release of calcium ions by the ER in oocytes, leading to ER stress and compromised developmental potential and fertilization capability. When the endoplasmic reticulum (ER) undergoes stress, on one hand, the intracellular calcium ion concentration increases, and oocytes would undergo appropriate calcium responses during fertilization and subsequent development for normal progression. Calcium ions also serve as crucial signaling molecules in cellular signal transduction. Excessive calcium influx can reciprocally affect the activity and function of the ER, leading to impaired protein synthesis and transport. Accumulation of unfolded and misfolded proteins further exacerbates ER stress [29]. Prolonged or severe ER stress can result in redistribution and structural changes in the ER, impacting normal physiological metabolism, inhibiting oocyte maturation, and even inducing cell apoptosis [30, 31]. On the other hand, the ER plays a critical role in the synthesis of cytoplasmic proteins, lipids, and secretory proteins, thus exerting a key function in oocyte metabolic renewal. ER stress disrupts the steady state of cytoplasmic substance synthesis, leading to impaired oocyte development [32].

Our study demonstrates that dysfunctional TUBB8 mutations lead to abnormalities in cytoskeletal structure, spindle assembly, and movement, resulting in oocytes that are only morphologically “mature” despite the expulsion of the first polar body during the first meiotic division. TUBB8, as a critical gene encoding structural protein, plays an important role in the regulation of microtubule dynamics during oocyte meiosis, maintenance of oocyte polarity, and assembly of the cytoskeleton during oocyte maturation. The identification of the described patients expands the spectrum of phenotypes observed in oocytes experiencing meiotic arrest due to TUBB8 mutations in two significant aspects. On one hand, deleterious mutations in TUBB8 directly affect the formation of microtubule proteins and the proper assembly of the spindle apparatus during oocyte meiosis [16]. On the other hand, aberrant chromosome distribution and organelle rearrangement in oocytes cause a decrease in nuclear-cytoplasmic maturation synchrony and a decline in developmental potential. Novel variants in the TUBB8 gene can affect spindle assembly, chromosome distribution, and organelle rearrangement during oocyte meiosis, leading to decreased nuclear-cytoplasmic maturation synchrony and oocyte maturation impairment.

In the present, we provide the evidence of the involvement of the microtubule synthesis system mediated by TUBB8 variants in oocyte maturation. Therefore, TUBB8 variant screening may serve not only as a genetic diagnostic marker for patients with oocyte maturation arrest but also have significant clinical implications for assessing patients’ oocyte developmental potential. It can provide precise molecular diagnosis and genetic counseling for patients with partial oocyte maturation disorders and lay the groundwork for future molecular therapies.

## Conclusion

In conclusion, this study establishes the pathogenicity of TUBB8 variants on oocyte maturation. Deleterious TUBB8 variants could disrupt microtubule function, thereby affecting critical processes such as spindle assembly, chromosome distribution, and organelle rearrangement during oocyte meiosis. These disruptions culminate in compromised nuclear-cytoplasmic maturation, consequently giving rise to oocyte maturation defects.

## Methods

### Selection of TUBB8 variants

All of selected TUBB8 variants in the present study have been identified by whole-exome sequencing and sanger sequencing from the primary female infertility patients with oocyte maturation arrest at the Reproductive Medicine Center of Xiangya Hospital of Central South University.

### Experimental animals

Female ICR mice (6 to 8 weeks old, SPF class) were procured from the Animal Research Center of Central South University, China. All mice were adapted for 3 days after their purchase and were maintained under a controlled temperature (26 ± 2 °C) with 12 h (h) light/dark conditions. All experiments were approved by the Ethics Committee of Xiangya Hospital of Central South University in China and the Institutional Animal Care and Use Committee (IACUC) of Central South University, in line with the welfare and ethical principles of laboratory animals.

### Immature oocytes collection

Ovaries were collected 48 h post-ovulation induction through intraperitoneal injection of 10 IU PMSG. Cumulus-oocyte complexes (COCs) were then collected in M2 medium. Germinal vesicle (GV) oocytes were isolated by carefully removing granulosa cells using a mouth-operated glass capillary pipette. Subsequently, the oocytes were maintained in a medium containing 2.5 µM milrinone to temporarily inhibit meiotic resumption, facilitating microinjection procedures via a micromanipulator.

### mRNA construct and in vitro transcription

Encoding full-length wild-type or mutant forms of TUBB8 in plasmid vectors containing a T7 promoter were constructed to drive expression. Wild-type (WT) TUBB8 complementary DNA (cDNA) was subcloned into pcDNA3.1/Flag vectors. Capped mRNA was synthesized from linearized plasmid using a T7 mMessage mMachine kit (Thermo Fisher Scientific, USA) and purified with a MEGAclear kit (Thermo Fisher Scientific, USA). Typically, 10 to 12 pl of mRNA (0.5 to 1.0 µg/µl) were injected into oocytes and then arrested at the GV stage in M16 medium containing 2.5 M milrinone for 4 h, allowing enough time for translation, followed by releasing into milrinone-free M16 medium and culturing at 37 °C in a humidified atmosphere of 6% CO2 for further study. Approximately 140 oocytes were injected in each group [33].

### Immunofluorescence and confocal microscopy

Oocytes were fixed in 4% paraformaldehyde for 30 min and permeabilized in 0.5% Triton X-100 for 20 min at room temperature. Then, oocytes were blocked with 1% bovine serum albumin (BSA)–supplemented phosphate-buffered saline (PBS) for 1 h and incubated with primary antibodies: DYKDDDDK Tag Antibody (Binds to same epitope as Sigma’s Anti-FLAG® M2 Antibody, 1:300 dilution, CST, 2368T), Alpha Tubulin Mouse Polyclonal antibody (1:300 dilution, Proteintech,66031-1-Ig) at 4 °C overnight. After washing in phosphate-buffered saline with Tween 20 (PBST), oocytes were incubated with an appropriate secondary antibody: CoraLite488-conjugated Goat Anti-Mouse(1:400dilution,Proteintech SA00013-1) and CoraLite594–conjugated Goat Anti-Rabbit(1:400dilution, Proteintech SA00013-4) for 1 h at room temperature. Then, oocytes were counterstained with Actin-Tracker Red-Rhodamin(Beyotime,C2207S) for 1 h or DAPI (Servicebio,G1012) for 10 min at RT, protected from light. Last, oocytes were mounted on glass slides and observed under a confocal microscope (ZEISS LSM 900, Germany). Approximately 50 oocytes were injected in each group [15, 33]. To quantify fluorescence intensity, signals from both control and treated oocytes were acquired through identical immunostaining procedures and confocal microscopy settings. The average fluorescence intensity per unit area within the region of interest was used to quantify the fluorescence in each oocyte image. Fluorescence intensity was randomly assessed through plot profiling using ImageJ software (National Institutes of Health, USA).

### Active mitochondrial staining

The oocytes were incubated with Mito Tracker Red CMXRos (1:1000 dilution, Beyotime, China) and 10 µg/ml Hoechst 33,342 in M2 medium. After washing with M2 medium three times, oocytes were mounted in the M2 medium droplet on the confocal dishes and observed by confocal microscopy (ZEISS LSM 900, Germany). Approximately 50 oocytes were used in each group [34]. Mito Tracker Red localization was detected by plot profile analysis with ImageJ software (National Institutes of Health, USA).

### Endoplasmic reticulum and Ca2 + staining

Oocytes were incubated in M2 medium with Pre-diluted ER-Tracker Red (1:1000 dilution, Beyotime, China) or Ca2^+^-sensitive probe Fluo-3 AM (1:1000 dilution, Beyotime, China) for 20 min at 37℃ in dark and then washed twice with M2 medium according to the manufacturer’s instructions. Oocytes were subsequently stained using 10 µg/ml Hoechst 33,342 in M2 medium. After washing with M2 medium three times, oocytes were mounted in the M2 medium droplet on the confocal dishes. Approximately 40 oocytes were used in each group [35, 36]. Red and Green immunofluorescence was respectively observed with LSM 900 confocal microscope. Results were analyzed using the ImageJ software (National Institutes of Health, USA) to measure the fluorescence intensity and distribution in each oocyte.

### Statistical analysis

Each experiment was conducted in triplicate, consistently yielding comparable results. The data, expressed as the mean ± standard deviation (SD), underwent statistical analysis using GraphPad Prism 9.0. For normally distributed numerical data, a two-way analysis of variance (ANOVA) was employed, with an assessment of homogeneity of variance. Differences between two or more rates were assessed using Chi-Square tests (χ2 test). A statistical significance threshold was set at *P* < 0.05.

## Data Availability

All data generated or analyzed during this study are included in this published article.
